# Bioactivity of Essential Oils from Patagonian Wild Plants: Acaricidal and Insecticidal Effects on *Varroa destructor* and *Apis mellifera*

**DOI:** 10.3390/plants14162484

**Published:** 2025-08-11

**Authors:** Rosa M. Manzo, Azucena E. Iglesias, Jimena J. Guajardo, Carolina A. Amaturi, Brenda D. Freeman, Juliana López de Armentia, Susana Rizzuto, Matías D. Maggi

**Affiliations:** 1 Consejo Nacional de Investigaciones Científicas y Técnica (CONICET), Buenos Aires CP 1425, Argentina; 2 Laboratorio de Investigaciones en Evolución y Biodiversidad, Universidad Nacional de la Patagonia San Juan Bosco-LIEB, Esquel CP 9200, Argentina; 3 Centro de Investigación en Abejas Sociales (IIPROSAM), Facultad de Ciencias Exactas y Naturales, Universidad Nacional de Mar del Plata, Mar del Plata CP 7600, Argentina; 4 Instituto Nacional de Tecnología Agropecuaria (INTA), Esquel CP 9200, Argentina; 5 Secretaria de Chubut, Rawson CP 9103, Argentina; 6 Instituto de Biotecnología Esquel, Universidad Nacional de la Patagonia San Juan Bosco-INBIES, Esquel CP 9200, Argentina

**Keywords:** organic control, honeybee, monoterpenes, varroosis, apiculture

## Abstract

*Varroa destructor* represents a major threat to honeybee colonies worldwide, prompting the search for alternative organic acaricides. This study evaluated the biological activity of essential oils extracted from three Patagonian wild plants—*Adesmia boronioides*, *Dysphania multifida*, and *Senecio filaginoides*—on both *V. destructor* and *Apis mellifera*. Chemical analysis revealed that *A. boronioides* oil was dominated by esquelenone (34.49%), *D. multifida* by ascaridole (34.87%), and *S. filaginoides* by *α*-pinene (40.87%). All essential oils exhibited acaricidal activity, with *D. multifida* showing the lowest LC_50_ against *V. destructor* (1.1 µL/mL at 24 h). Toxicity assays on adult bees indicated that *A. boronioides* and *D. multifida* significantly reduced bee survival, whereas *S. filaginoides* did not appear to cause significant mortality (LC_50_ = 139.5 µL/mL). Repellency tests for *A. boronioides* and *D. multifida* showed significant mite repellence. Larval assays revealed a high survival rate under *S. filaginoides* treatment (survival rate > 80.24%), in contrast to the reduced viability observed with the other oils. The high selectivity index of *S. filaginoides* underscores its potential as a selective and safe botanical acaricide. Moreover, its LC_50_ decreased over time, suggesting a residual acaricidal effect. These findings support *S. filaginoides* as a promising candidate for sustainable *V. destructor* control.

## 1. Introduction

*Apis mellifera* is a globally relevant species due to, on the one hand, its role as a pollinator of flowering plants—an essential component of many agricultural systems—and, on the other hand, the valuable hive products it provides (e.g., honey, propolis, royal jelly) for the beekeeping industry [1,2]. However, *Apis mellifera* is susceptible to pathogens and parasites, leading to significant economic losses in both the beekeeping industry and agriculture due to the decline in numerous colonies [1,3,4,5]. Among these threats, *Varroa destructor* [6] is recognized as the most detrimental parasite of managed honeybee colonies worldwide [7]. Several factors contribute to the impact of *V. destructor* on honeybee populations, including the damage it causes by feeding on immature and adult bees, its role as a confirmed vector of various debilitating viruses (and potentially others that exacerbate its impact on bee health), and its ability to inhibit the immune responses of parasitized bees [7,8,9,10,11,12]. Although *V. destructor* infests both adult and immature bees, its reproduction occurs exclusively within brood cells [13]. After emergence, the female mite enters a dispersal phase during which it resides on adult bees, feeding mainly on body fat tissues [14,15]. This parasitism can lead to severe damage and, in extreme cases, colony collapse [7,13,15].

Traditionally, beekeepers have relied on a range of synthetic acaricides, including organophosphates and pyrethroids [16,17,18,19,20], but this approach presents critical limitations. These chemical substances often leave residual contaminants in hive products, which are later sold for human consumption, raising significant food safety concerns [21]. Additionally, the widespread and improper use of these acaricides has imposed selective pressures on mite populations, leading to the development of resistance to several commonly used treatments, such as Coumaphos, Amitraz [17,18], and Flumethrin [16,19]. Consequently, there is growing interest in developing eco-friendly alternatives, particularly organic acaricides derived from botanical products [22,23,24,25].

Argentine Patagonia hosts a diverse flora, including many native species traditionally used by the local population that are rich in biologically active compounds [26]. Numerous plant-derived substances have demonstrated physiological and behavioral effects against insect pests, and they can serve as new sources for the development of natural pesticides [27,28,29]. Due to the urgent need for green and sustainable pesticides, this study focuses on plants growing in this promising region: Paramela (*Adesmia boronioides* Hook.f.), Paico (*Dysphania multifida* (L.) Mosyakin y Clemonts), and Senecio (*Senecio filaginoides* DC.). All of these are recognized for their aromatic and medicinal properties in Patagonian traditional medicine and culture and are of great scientific interest due to their numerous biological activities [30]. *A. boronioides* is a resinous shrub, 0.40 to 2 m high [31]. It is an aromatic and medicinal species belonging to the Fabaceae family [30]. This species has received increasing attention, especially for its essential oil, which has been reported to have antimicrobial, antifungal, trypanocidal, and anti-inflammatory properties [32]. *D. multifida* is an aromatic and annual herb covered with oil-secreting hairs [33]. Its essential oils have shown potentially antibacterial, antifungal, and insecticidal effects [34]. *S. filaginoides* is a shrub belonging to the Asteraceae family [35]. In traditional medicine, it has been used for its antimicrobial, antifungal, and insecticidal properties [35].

Given the urgent need for environmentally sustainable acaricides, this study aims to assess the biological effect of essential oils (EOs) from *Adesmia boronioides*, *Dysphania multifida*, and *Senecio filaginoides* on *V. destructor* and *A. mellifera* (adults and larvae). Specifically, the objectives are to (1) determine the LC_50_ values of these essential oils for both *Varroa destructor* and *Apis mellifera*, (2) evaluate their potential toxicity on *A. mellifera* larvae, and (3) assess their attractive and repellent effects on *V. destructor*.

## 2. Results

### 2.1. Chemical Analysis: Constituents of Essential Oils

The essential oils exhibited distinct compositional profiles. Esquelenone (34.49%) was the most abundant compound in the EO of *A. boronioides*, ascaridole (34.87%) was predominant in the EO of *D. multifida*, and *α*-pinene (40.87%) was the major constituent in the EO of *S. filaginoides*, as listed in Table 1.

### 2.2. Mite and Bee Bioassays

In all cases, the estimated LC_50_ value at 24 h was lower for *V. destructor* than *Apis mellifera*. The EO of *D. multifida* showed the lowest estimated LC_50_ value against *V. destructor*, as shown in Table 2. Similarly, *D. multifida* also had the lowest estimated LC_50_ value for *A. mellifera*, as shown in Table 3.

The selectivity index (LC_50_ *A. mellifera*/LC_50_ *V. destructor*) at 24 h was 25 for *A. boronioides*, 3.1 for *D. multifida*, and >100 for *S. filaginoides*.

Bee mortality was significantly higher following the application of essential oils from *A. boronioides* and *D. multifida* compared to the control (NOAEL of 5 μL/mL (X2 (1, N = 50) = 8, *p* = 0.004), and NOAEL of 0.5 μL/mL (X2 (1, N = 50) = 18.1, *p* = 0.00002), respectively). However, no significant difference in bee mortality was observed between the treatment and control groups for *S. filaginoides* (NOAEL of 100 μL/mL (X2 (1, N = 50) = 0.35, *p* = 0.555)).

### 2.3. Attraction and Repellency Assay

Essential oils from all tested plant species showed mixed effects on *Varroa destructor* attraction and repellency. The binomial test on expected mite location within the Petri dish revealed that no significant difference was observed between the treated and control sides for *Senecio filaginoides* (*p* = 1.00) and the control group (*p* = 0.581). However, a significant repellency effect was detected for *Dysphania multifida* (*p* = 0.022) and *A. boronioides* (*p* = 0.001), as shown in Table 4.

### 2.4. Toxicity Bioassay on Apis mellifera Larvae

**Figure 1:**
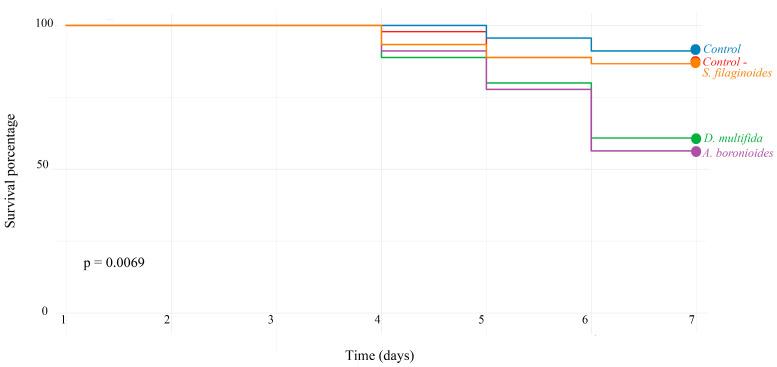
Kaplan–Meier survival curves of honeybee larvae under different treatments: Control-, Control, and essential oils of *A. boronioides*, *D. multifida*, and *S. filaginoides*. The global log-rank test indicated a significant difference among the survival curves (*p* = 0.0069).

By the sixth day, honeybee larvae survival was lowest for the essential oil of *A. boronioides* at 52.05%, followed by *D. multifida* (56.26%) and *S. filaginoides* (80.24%). Meanwhile, the controls showed survival rates above 80%, as seen in Figure 1. The log-rank test for the overall comparison among all groups yielded a significant result (*p* = 0.0069), indicating differences in survival distributions. Subsequently, based on pairwise comparisons using the Cox proportional hazards model, larvae exposed to the essential oils of *Adesmia boronioides* (*p* = 0.019) and *Dysphania multifida* (*p* = 0.009) showed a significantly increased risk of mortality compared to the control group. No significant effect was observed for *Senecio filaginoides* and the negative control group (acetone only), as shown in Table 5.

## 3. Discussion

This study provides new evidence on the acaricidal potential of three aromatic plant species from Patagonia—*Adesmia boronioides*, *Dysphania multifida*, and *Senecio filaginoides*—as natural sources of bioactive compounds for the control of *Varroa destructor* in *A. mellifera*. The essential oils extracted from these species demonstrated significant lethality against the mite, with LC_50_ values comparable to those reported in previous studies from South America [18,20,29,36]. For example, Ruffinengo et al. [36] identified *Acantholippia seriphioides* as one of the most potent species in their study, with an LC_50_ of 1.2 µL/mL—comparable to the LC_50_ of *A. boronioides* (1.1 µL/mL) observed in our study. However, an even lower LC_50_ was found for *D. multifida*, which exhibited a value of 0.3 µL/mL.

Notably, the LC_50_ of *S. filaginoides* decreased markedly over time, from 139.5 µL/mL at 24 h to 9.1 µL/mL at 72 h, suggesting a residual effect whereby lower concentrations achieve equivalent mite mortality after extended exposure. This prolonged efficacy could reduce the frequency of applications under field conditions and may help delay the development of resistance to botanical acaricides. However, this pattern warrants further investigation, particularly regarding the residue dynamics of the *S. filaginoides* essential oil within the hive environment. Monitoring potential residues in honey, beeswax, pollen, soil, and water is essential to assess both food safety and environmental risks [37].

Our assays demonstrated that the essential oils of *A. boronioides* and *D. multifida* induced repellent effects on *V. destructor*, whereas *S. filaginoides* elicited no significant attraction or repellency. These findings contrast with those of Iglesias et al. [20], who observed no behavioral responses in mites exposed to *Humulus lupulus* L. essential oils. However, this discrepancy aligns with the broader literature highlighting substantial variability in the behavioral responses of *V. destructor* to volatile compounds [23,36,38]. It is well established that the pit organ located on the forelegs of female *V. destructor* functions as the primary sensory structure for host orientation, selection, and reproductive behaviors, playing a pivotal role in their survival [39,40]. This organ is responsible for detecting environmental chemical cues and is therefore likely to mediate behavioral responses to volatile compounds [41].

In our study, the major compounds identified in the repellent essential oils were ascaridole and esquelenone. Ascaridole, a monoterpene predominant in *D. multifida*, has been associated with high toxicity [42]. Similarly, esquelenone, a sesquiterpene present in *A. boronioides*, exhibits toxicity against other arthropods [29], and our findings provide further evidence of its efficacy as a repellent. The repellent effects of these compounds may stem from their interference with *Varroa destructor*’s chemosensory perception. The pit organ, which responds to volatile stimuli [41], is likely disrupted by these compounds, impairing the mite’s ability to detect host-derived cues. This disruption of host recognition mechanisms could explain the repellent effects observed in our assays. However, further studies are required to elucidate the specific molecular targets and behavioral outcomes associated with these compounds in *V. destructor*.

Evaluating larval survival is essential for assessing the safety of essential oils as acaricidal treatments in beekeeping, as their use in hives must not compromise brood development. In our assays, only *S. filaginoides* exhibited high larval survival (80%), comparable to control groups, while *A. boronioides* and *D. multifida* significantly reduced survival rates to 52% and 56%, respectively, indicating a higher toxicity to larvae. Although all tested oils exhibited effective acaricidal activity, only *S. filaginoides* combined this efficacy with a favorable safety profile for bee larvae.

On the other hand, the NOAEL (No Observed Adverse Effect Level) analysis in adult bees revealed notable differences in toxicity among the tested essential oils. *S. filaginoides* displayed a non-significant NOAEL, whereas both *A. boronioides* and *D. multifida* exhibited toxicity at low concentrations (5 µL/mL and 0.5 µL/mL, respectively), indicating a narrower safety margin. This information is essential when selecting plant-derived compounds for field application, particularly considering a recurrent limitation in the use of essential oils: the narrow margin between the LC_50_ values for *V. destructor* and those for honeybees [25,43,44]. In this regard, *S. filaginoides* showed a selectivity index of >100, suggesting a remarkably wider safety margin compared to *A. boronioides* (25) and *D. multifida* (three).

Plants continuously synthesize and accumulate a wide array of secondary metabolites which, although not directly involved in primary physiological processes, serve as a rich source of bioactive compounds with potential applications in crop protection and pest management [45,46]. In the case of *S. filaginoides*, *α*-pinene (40.87%) and *β*-pinene (32.96%) were identified as major constituents, consistent with previous studies [47]. Both monoterpenes have demonstrated acaricidal effects against *Rhipicephalus microplus* (Canestrini, 1888) [48,49]. Additionally, *α*-pinene has shown greater toxicity than the essential oil of *Rosmarinus officinalis* L. against the two-spotted spider mite, *Tetranychus urticae* Koch, 1836 [50]. These findings align with studies indicating that several monoterpenes exert neurotoxic effects in mites [51].

In addition to the dominant pinenes, the essential oil of *S. filaginoides* contained sabinene and limonene at relatively lower concentrations (5.40% and 4.12%, respectively). Although these compounds were detected using GC-MS with nitrogen as the carrier gas and a column with 1.0 µm film thickness—conditions suitable for major compound identification—future analyses could consider the use of helium or hydrogen and thinner film columns (≤0.32 µm) to improve resolution for minor constituents [52], particularly since these compounds could contribute to the oil’s overall bioactivity. Among these, sabinene is a bicyclic unsaturated monoterpene found in the essential oils of various plant species [53] and is known for its antibacterial and antifungal properties [54,55]. Similarly, limonene has been reported to exert acaricidal effects against the stored product mite *Tyrophagus putrescentiae* (Schrank, 1781) and other arthropods [56]. As emphasized by Bava et al. [57], the biological activity of essential oils often results from complex interaction and synergism among multiple components rather than a single dominant compound. Therefore, further investigation is required to determine the individual and synergistic contribution of the essential oil’s constituents to its overall acaricidal activity and support their relevance as active components in mite control strategies.

Overall, our study highlights the potential of regional flora, particularly *S. filaginoides* essential oil, as a sustainable biopesticide, supporting the development of locally sourced, eco-friendly alternatives for apicultural pest management. While laboratory results show promising acaricidal activity with low larval toxicity, translating these findings into practical applications requires overcoming the inherent challenges of the apiary environment. Factors such as temperature, humidity, colony dynamics [57], and the natural volatility and degradation of active compounds [37] may limit its efficacy. Given these constraints, we emphasize that rigorously controlled field trials are an indispensable next step to validate the utility of *S. filaginoides* EO under real-world beekeeping conditions. Such trials must account for ecological variables (e.g., colony strength, climate, foraging activity) and biological complexities (e.g., mite resistance dynamics, non-target effects) to ensure both scalability and long-term effectiveness. To this end, we are currently designing multidisciplinary field studies that integrate acaricidal efficacy monitoring, hive health assessments—particularly regarding potential sublethal effects on brood, queens, and hive microbiota—and formulation optimization.

## 4. Materials and Methods

### 4.1. Collection of Plants and Extraction of Essential Oils (EOs)

Natural plant populations located in the northwestern region of Chubut province were selected. Plant material was collected during the pre-flowering phenological stage, as this period is typically associated with the highest concentration of phenolic compounds in Patagonian plants [42]. Only the branches of each plant species were harvested to preserve the plants, with the total weight ranging from 800 g to 1000 g. *Adesmia boronioides* was collected from populations located at the coordinates −42.856930, −71.287078, *Dysphania multifida* from −42.930371, −71.364734, and *Senecio filaginoides* from −42.910075, −71.342718.

The essential oils were obtained through steam distillation using the equipment available at the Essential Oils Extraction Unit (UEAE) of the Esquel Biotechnology Institute (INBIES), part of the National University of Patagonia San Juan Bosco (UNPSJB). This process was conducted on a pilot scale, employing a 4-liter pilot distillation apparatus. For extraction, the phytomass of each species was distilled with saturated steam at 100 °C for 1 h and 30 min. Multiple distillations were performed until the required volume was obtained to be able to carry out all bioassays. The yield of each distillation was calculated and expressed as the volume (mL) of essential oil obtained per kilogram of processed phytomass (kg). The essential oils obtained were stored in amber glass bottles, sealed and kept at 4 °C, and protected from sunlight until use.

### 4.2. Analysis of Essential Oils

The essential oils obtained were injected into a gas chromatograph to obtain chromatograms and determine chemical composition. Initial experiments using flame ionization detection (FID) were conducted to optimize separation, testing columns with different stationary phases and temperature programs. After optimization, samples were analyzed using a gas chromatograph coupled with a mass spectrometer (GC-MS), specifically a CHROMPACK CP-9003 gas chromatograph equipped with a CP-SIL 5CB-MS FS capillary column (60 m × 0.32 mm, film thickness 1.0 µm), with nitrogen as the carrier gas. The temperature program started at 60 °C (0 min hold), increasing at 4 °C/min to 240 °C. The injector temperature was 270 °C, and detection was performed with FID at 270 °C. Identification of major compounds was achieved by comparison with mass spectrum libraries available at UEAE-INBIES.

### 4.3. Source of Mites and Bees

*A. mellifera* workers and larvae, as well as adult females of *V. destructor*, were obtained from the experimental apiary of the Universidad Nacional de la Patagonia San Juan Bosco (42°55′52.8″ S 71°21′48.9″ W) during the summer and fall of the 2023–2024 season. Colonies with less than 1% of mite infestation in adult worker bees were considered healthy and were used as a source of bees (adults and larvae) for the bioassays. Colonies with more than 5% infestation in adult bees were considered highly infested and were selected as the source of *Varroa destructor* for the trials. From these colonies, combs with operculated broods from at least three different hives were selected and taken to the laboratory for the collection of adult female mites, selecting only those with a dark brown coloration. Nurse worker bees were collected from combs with an open brood in at least three healthy colonies.

### 4.4. Experimental Design for Mite and Bee Bioassays

**Figure 2:**
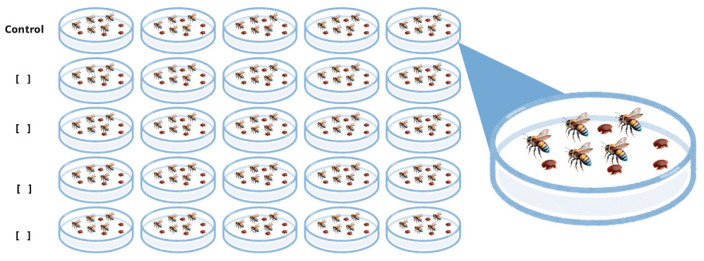
Schematic representation of the bioassay. The procedure was repeated for each essential oil using its corresponding concentrations.

The bioactivity levels of the essential oils from *Adesmia boronioides*, *Dysphania multifida*, and *Senecio filaginoides* were assessed using the complete exposure method [36]. The treatments were conducted in 10 × 10 cm Petri dishes, using a new dish for each test. Each essential oil was diluted in acetone to the desired concentration based on the previous assay: *Adesmia boronioides*: 0.5, 1, 1.5, 2.5, 5, 10, and 20 μL/mL per capsule; *Dysphania multifida*: 0.5, 1, 1.5, 2.5, 5, 10, and 20 μL/mL per dish; and *Senecio filaginoides*: 5, 10, 20, 25, 50, and 100 μL/mL per dish. Then, 1 mL of each prepared solution was applied to the bottom of the Petri dishes. Five replicates were performed per treatment, along with a single control using 1 mL of acetone, as shown in Figure 2. After application, the plates were left open for 5 min to allow the solvent to evaporate. Once evaporation was completed, five newly emerged adult bees (0 to 3 days old) collected from healthy bee colonies and five adult female *V. destructor* mites, obtained from capped brood cells of highly infested colonies, were placed into the dishes. The bees and mites were exposed to the treatments for 72 h, and mortality rate was recorded at 24, 48, and 72 h. Bees were provided with 3 g of candy (a mixture of water and powdered sugar) and water-moistened sponges to maintain humidity. During the trial, the dishes were kept in an incubator at 30 °C and 60% relative humidity. Mite and bee mortality was assessed at each time point by direct observation using a magnifying lens. Additionally, the selectivity index (calculated as LC_50_ *A. mellifera*/LC_50_ *V. destructor*) was determined for each treatment and each observation time.

### 4.5. Attractive and Repellent Assay

**Figure 3:**
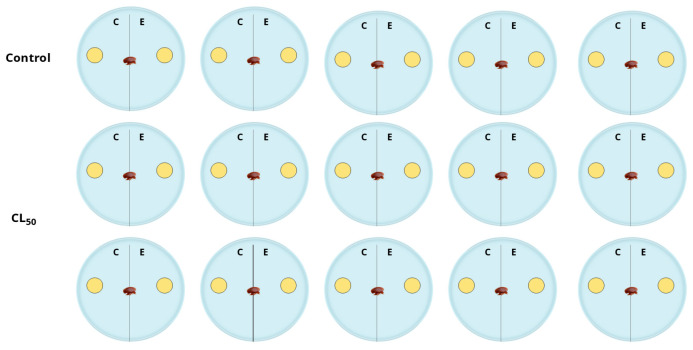
Schematic representation of the attraction and repellency bioassay. The assay was repeated for each essential oil using the corresponding LC_50_.

The methodology was adapted from Damiani et al. [23]. Each Petri dish (10 cm in diameter) was divided into two sections (C and E), as seen in Figure 3. In section C, a 1 cm diameter filter paper disc was placed and impregnated with 8 μL of the LC_50_ concentration previously calculated for each essential oil. In section E, another filter paper disc impregnated with acetone (used as a solvent) was placed. For the control group, only a disc impregnated with acetone was placed in section C, while section E remained empty.

After allowing the solvent to evaporate, a single adult *V. destructor* female was introduced at the center of each Petri dish. After 90 min, the location of the mite (C or E) was recorded. Each essential oil and control group was tested in thirty replicates, as in Figure 3.

### 4.6. Honeybee Larval Toxicity Bioassay

**Figure 4:**
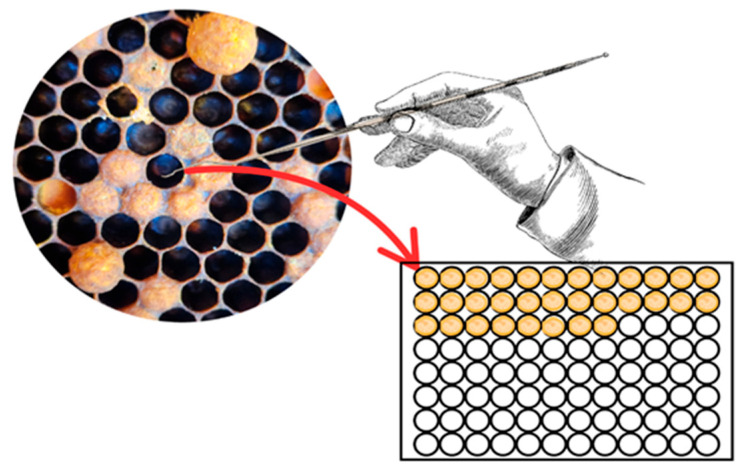
Schematic representation of the larval bioassay. L1 larvae were grafted from brood combs and placed in cell culture well plates in groups of 35 individuals. The procedure was repeated for each treatment group.

One-day-old larvae (L1) were meticulously collected from the brood cells of healthy bee colonies and transferred (grafted) into 48-well cell culture plates, following the methodology described by Ramírez et al. [58]. For each assay, five plates (one per treatment group) were prepared. Each plate contained 35 larvae, forming a plate/group, as shown in Figure 4. Three plates were assigned to the essential oil treatments (each using the LC_50_ concentration of *A. boronioides*, *D. multifida*, or *S. filaginoides*), one plate was assigned as a negative control (acetone only), and one plate as a control without acetone or essential oil —totaling five plates/group and 175 larvae per assay.

The assay was repeated three times at different time intervals, using larvae from different bee colonies. Larvae were fed a basic larval diet according to the protocol by Aupinel et al. [59]: 10 μL daily during the first two days, followed by 20, 30, 40, and 50 μL on days 3, 4, 5, and 6, respectively. Starting on day 3, essential oil treatments were administered by supplementing the diet with 1 μL of the corresponding LC_50_ concentration. Control groups received either acetone or no additive. Acetone was used as a solvent due to its low toxicity in bee larvae [60]. The plates were then placed into a desiccator maintained at 96% relative humidity (using saturated K_2_SO_4_) in an incubator set at 34 °C. The experiment was independently replicated three times to minimize handling errors.

Larval survival was monitored daily using a stereoscope, with spiracle movement (opening and closing) used as an indicator of vitality. Larvae were considered dead if no movement was detected. The survival rate was calculated following Dai et al. [61]: (number of larvae reaching day 6 (D6)/number of larvae grafted at the start of the experiment) × 100.

### 4.7. Statistical Analysis

Mite and bee mortality rates were assessed by visual inspection of the Petri dishes at 24, 48, and 72 h post-exposure. Median lethal concentrations (LC_50_) values and their corresponding 95% confidence intervals for *V. destructor* were estimated using logistic regression (PROC LOGISTIC) [62,63]. For *Apis mellifera*, the highest essential oil concentration that did not produce a statistically significant bee mortality level compared to controls (No Observed Adverse Effect Level, NOAEL (*p* = 0.05)) was estimated following the methodology proposed by Medrzycki et al. [60]. Statistical differences between uncorrected mortality in the treatment groups were analyzed using the Chi-squared (x^2^) test.

To assess the attraction or repellency of *Varroa destructor* to essential oils, a binomial test was used to compare the observed number of mites on the stimulus side (section C) versus the opposite side (section E), excluding mites located in the neutral sectors. This analysis tested whether mites were significantly attracted to or repelled by the stimulus (α = 0.05). Additionally, Fisher’s exact test was applied to compare the distribution of mite positions in the treatment groups versus the control group, in order to evaluate whether the essential oils induced a different spatial response compared to acetone alone. All statistical analyses were performed in R [64].

To evaluate the effect of essential oils on larval survival over time, Cox proportional hazards models were used. Survival data from all three trials were pooled, and a treatment group was included as a fixed effect. The analysis was performed using the coxph() function from the survival package in R [65]. The proportional hazards assumption was assessed using Schoenfeld residuals via the cox.zph() function. Hazard ratios (HRs) and their 95% confidence intervals were calculated to compare the risk of mortality among treatments, using the positive control group as the reference. Statistical significance was set at α = 0.05.

## Figures and Tables

**Table 1:** Major constituents of EOs of *A. boronioides*, *D. multifida*, and *S. filaginoides*. Only compounds representing >0.2% are listed.

Compound	*A* *. boronioides*		*D. multifida*		*S. filaginoides*	
	R.T. ^1^	(%)	R.T.	(%)	R.T.	(%)
Monoterpene						
Tricyclene					22.08	0.611
*α*-Pinene	22.55	11.41	22.62	0.339	22.65	40.872
Camphene					23.52	0.333
Sabinene					24.27	5.403
Myrcene					24.58	1.255
*β*-Pinene	24.60	1.95	24.69	0.246	24.78	32.962
*α*-Terpinene			25.55	0.192	26.08	0.490
*δ*-3-Carene			26.13	26.05	26.28	0.871
Limonene	26.63	3.18			26.83	4.126
*Z*-*β*-Ocimene			26.71	0.199		
*β*-Phellandrene					27.01	2.506
*γ*-Terpinene			27.85	0.219	27.85	0.265
Linalool					28.30	0.35
Terpinolene					29.16	0.988
4-Terpineol					33.47	0.847
Citronellol					34.59	0.386
Ascaridole			36.30	34.87		
Piperitone epoxide			38.40	8.26		
Alkylbenzene						
*p*-Cymene			26.28	24.39		
Sesquiterpene						
*β*-Caryophyllene			37.54	3.65		
Guaiadiene	44.23	10.23				
*10-epi-γ*-Eudesmol	49.13	7.22				
Esquelenone	46.17	34.49				
*β*-Furopelargane	46.90	3.57				
*α*-Furopelargane	47.28	2.08				
Isoesquelenone	50.28	10.61				
*4-α*-Dihydroagarofuranol	51.81	5.77				

^1^ R.T.: Retention time of the essential oil compound in the chromatographic column.

**Table 2:** Estimated LC_50_ values for *V. destructor* at each time interval for each essential oil. Lower and upper confidence limits are shown in parentheses.

Essential Oil		*Varroa destructor* LC_50_ (µL/mL)	
	24 h	48 h	72 h
*Adesmia boronioides*	1.1 (0.79–1.56)	0.28 (0.16–0.50)	>0.5
*Dysphania multifida*	0.31 (0.02–1.91)	0.95 (0.26–3.38)	>0.5
*Senecio filaginoides*	139.5 (69.2–280.1)	78.6 (32.4–190.2)	9.1 (5.4–14.8)

**Table 3:** Estimated LC_50_ values for *A. mellifera* at each time interval for each essential oil. Lower and upper confidence limits are shown in parentheses.

Essential Oil		*Apis mellifera* LC_50_ (µL/mL)	
	24 h	48 h	72 h
*Adesmia boronioides*	28.02 (11.01–71.33)	13.1 (6.64–25.9)	14.4 (5.9–35.2)
*Dysphania multifida*	0.98 (0.5–1.75)	0.94 (0.25–3.46)	>20
*Senecio filaginoides*	>10000	>1000	>1000

**Table 4:** Attraction and repellency effect of *V. destructor* in response to essential oils (a binomial test).

Essential Oil	Effect	*p*-Value
*Control*	No effect	0.581
*Adesmia boronioides*	Repellency	0.001 **
*Dysphania multifida*	Repellency	0.022 *
*Senecio filaginoides*	No effect	1.00

Asterisks indicate significance levels: * denotes *p* < 0.05; ** denotes *p* < 0.01.

**Table 5:** Cox proportional hazards model for larval survival. Hazard ratios (exp (coef)), confidence interval standard errors (lower–upper), and corresponding *p*-values are shown.

Treatments	HR (exp(coef))	95% Confidence Interval	*p*-Value
*Control-*	1.53	0.43–5.42	0.509
*Adesmia boronioides*	3.745	1.23–11.38	0.019 *^1^
*Dysphania multifida*	4.299	1.43–12.86	0.009 **
*Senecio filaginoides*	1.541	0.43–5.46	0.502

^1^ Asterisks indicate significance levels: * denotes *p* < 0.05; ** denotes *p* < 0.01.

## Data Availability

The original contributions presented in this study are included in the article. Further inquiries can be directed to the corresponding authors.
